# Anti-oxidative Amino Acid L-ergothioneine Modulates the Tumor Microenvironment to Facilitate Adjuvant Vaccine Immunotherapy

**DOI:** 10.3389/fimmu.2019.00671

**Published:** 2019-04-04

**Authors:** Sumito Yoshida, Hiroaki Shime, Misako Matsumoto, Masanori Kasahara, Tsukasa Seya

**Affiliations:** ^1^Department of Vaccine Immunology, Graduate School of Medicine, Hokkaido University, Sapporo, Japan; ^2^Department of Pathology I, Graduate School of Medicine, Hokkaido University, Sapporo, Japan; ^3^Department of Immunology, Graduate School of Medical Sciences, Nagoya City University, Nagoya, Japan; ^4^Department of Immunology, School of Pharmaceutical Sciences, Aomori University, Aomori, Japan

**Keywords:** tumor microenvironment, tumor-associated macrophage, cytotoxic T lymphocyte, toll-like receptor, L-ergothioneine

## Abstract

Cancer vaccines consist of a tumor-associated antigen (TAA) and adjuvant. These vaccines induce and activate proliferation of TAA-specific cytotoxic T lymphocytes (CTLs), suppressing tumor growth. The therapeutic efficacy of TAA-specific CTLs depends on the properties of tumor microenvironment. The environments make immunosuppressive by function of regulatory T cells and tumor-associated myeloid cells; thus, regulation of these cells is important for successful cancer immunotherapy. We report here that L-ergothioneine (EGT) with the adjuvant Toll-like receptor 2 (TLR2) ligand modulated suppressive microenvironments to be immune-enhancing. EGT did not augment DC-mediated CTL priming or affect CTL activation in draining lymph node and spleen. However, EGT decreased the immuno-suppressive function of tumor-associated macrophages (TAMs). TLR2 stimulation accompanied with EGT administration downregulated expression of PD-L1, CSF-1R, arginase-1, FAS ligand, and TRAIL in TAMs, reflecting reduction of CTL suppression. An anti-oxidative thiol-thione residue of EGT was essential to dampening CTL suppression. The effect was specific to the thiol-thione residue of EGT because no effect was observed with another anti-oxidant N-acetyl-L-cysteine (NAC). A CTL-suppressive environment made by TLR2 is relieved to be improved by the addition of EGT, which may ameliorate the efficacy of vaccine immunotherapy.

## Introduction

Cancer immunotherapy has therapeutic merit as a treatment for progressive cancers. Cytotoxic T lymphocytes (CTLs) are a key effector inducing tumor cell lysis. Tumor-specific antigen (TAA) uptake and cross presentation by dendritic cells foster T-cell priming in lymph nodes. CTLs proliferate and migrate to tumors, where they encounter tumor cells. Anti-PD-1 Ab treatment showed that TAA-specific CTLs regress progressive tumors in some patients. However, CTL function is usually impaired in the tumor microenvironment of progressive cancers (1, 2). This finding suggests that improvement of the environment is indispensable for achieving complete remission in immunotherapy.

Several myeloid subsets create immuno-suppressive microenvironments in the tumor stroma. Tumor-associated macrophages (TAMs) are representative subsets that suppress CTLs in tumors. Suppression has been explained by several mechanisms: (i) production of reactive oxygen and nitrogen species (ROS/RNS): peroxynitrite, a complex of ROS and RNS, inhibits CTL functions via nitration to cause dysfunction of T cell receptors (TCRs) and IL-2 receptors; (ii) expression of inhibitory signal mediators/stimulators such as IL-10, TGF-β, and PD-L1; (iii) depletion of nutrition via arginase-1 expression; and (iv) CTL apoptosis induced by FAS ligand and TRAIL on TAMs (3, 4).

Myeloid cells including dendritic cells and macrophages express Toll-like receptor 2 (TLR2) (5). TLR ligand acts on DCs as an adjuvant of cancer vaccines because it induces maturation and cross presentation of DCs (5). Cross presentation provokes the priming of CTLs in concert with extracellular soluble antigens and MHC class I (6) and induces tumor-responsive CTLs in combination with TAA (5, 7). Human CD141^+^ DCs, a major subset of DCs for cross presentation, abundantly express TLR2 heterodimers (TLR 2/1 and 2/6) (8), suggesting that TLR2 ligand might be a promising adjuvant for cancer vaccines. Our study and others showed that a number of TLR ligands facilitate cancer immunotherapy (9–11) mainly to increase CTLs in tumor environment.

Administration of TLR2 ligand also modulates the tumor microenvironment to induce immune suppression (12, 13). Upon TLR2 stimulation, macrophages in tumors activated, and promote tumor progression and invasion (14, 15). In this context, the intrinsic TLR2 ligand versican promotes tumor progression (14) and the tumor-derived TLR2 ligand hyaluronan induces formation of immunosuppressive macrophages (15). Hence, TLR2 ligand acts on both DCs and TAMs to positively or negatively modulate the microenvironment.

We have investigated applicability of L-ergothioneine (EGT) to excluding the immune-suppressive activity of macrophages. We previously reported that EGT has no immune stimulatory function, but up-regulates TLR responses in bone marrow-derived macrophages, which leads to strong innate immune activation (16). EGT is an anti-oxidative thiol derivative of L-histidine, and naturally synthesized by bacteria and fungi. EGT exists as a thiol-thione tautomer in physiological conditions (17). The tissue distribution of EGT depends on the expression profile of transporter OCTN1 (18, 19). Myeloid cells but not lymphocytes in peripheral blood abundantly express OCTN1 (20), and the biased expression of OCTN1 is involved in non-uniformal EGT distribution (21). EGT has been reported from *in vitro* studies to be an anti-oxidant, anti-inflammatory and cytoprotective activities (17) in addition to the innate immune-enhancing function (16). Yet, no *in vivo* studies have proved that EGT actually participates in tumor regression. Thus, it has been intriguing what happens in tumor-loading mice when they are treated with EGT together with TAA + TLR2 adjuvant.

Here, we show that EGT successfully improves *in vivo* therapeutic efficacy of TLR2 ligand/TAA via controlling TAM function.

## Materials and Methods

### Reagents

L-ergothioneine and L-hercynine were purchased or kindly provided by Tetrahedron (Paris, France). N-acetyl-L-cysteine (NAC) was purchased from Sigma-Aldrich (St. Louis, MO, USA). Ovalbumin (OVA)_257−264_ peptide (SIINFEKL,SL8 peptide) was purchased from MBL (Nagoya, Japan). OVA protein used in this study was high grade of immunochemistry: EndoGrade Ovalubmin (Hyglos, Bernried, Germany) and Ovalbumin, Low Endotoxin (Wako, Tokyo, Japan). 2,3-bis(palmitoyl) propyl Cys-Ser-Lys-Lys-Lys-Lys (Pam2CSK4) was synthesized by Biologica Co. Ltd (Nagoya, Japan). PBS used to substrate solution or injection into mice was endotoxin-free grade (Merck, Darmstadt, Germany). Antibodies (Abs) used for magnetic sorting were CD8 MicroBeads and Streptavidin MicroBeads (Miltenyi Biotec, Bergish Gladbach, Germany). The H2K^b^-SL8 peptide tetramer was utilized for measuring the levels of OVA-specific CTL (22, 23). We used the authentic beads purchased from MBL. Other Abs used in this study are mostly commercially available and listed in Supplementary Table 1.

### Mice

Inbred female wild-type C57BL/6 mice were purchased from Clea Japan. Mice were maintained under specific pathogen-free conditions and used in the age of 7–11 weeks. All animal experiments were approved by the Institutional Animal Care and Use Committee of Hokkaido University (the number was 18-0032) and performed in compliance with their guidelines.

### Cell Culture

Ovalbumin-expressing Lewis lung carcinoma (LLC-OVA) cells (22) were kindly provided by Dr. T. Nishimura and Dr. H. Kitamura (Hokkaido University). LLC-OVA cells were cultured in Iscove's Modified Dulbecco's Medium (IMDM, purchased from Thermo Fisher Scientific, Waltham, MA, USA) supplemented with 10% heat-inactivated fetal bovine serum (FBS, purchased from GE Healthcare Life Sciences, Buckinghamshire, England), 2 mM of L-glutamine (Thermo Fisher Scientific), 25 mM of HEPES buffer (Thermo Fisher Scientific), 55 μM of 2-ME (Thermo Fisher Scientific), 100 U/mL of penicillin-streptomycin (Thermo Fisher Scientific), and 100 μg/mL of G418 (Roche, Basel, Switzerland, purchased from Sigma-Aldrich). OVA-positive EG7 lymphoma cells (ATCC® CRL-2113™) were purchased from ATCC (Manassas, VA, USA) and cultured in RPMI 1640 supplemented with 10% heat-inactivated FBS, 10 mM of HEPES, 1 mM of sodium pyruvate, 55 μM of 2-mercaptoethanol, 100 IU of penicillin/100 μg/mL of streptomycin and 500 μg/mL of G418. The properties of this lymphoma line were mentioned in earlier papers (24). Immune cells harvested from mice were cultured in RPMI1640 (Thermo Fisher Scientific) supplemented with 10% heat-inactivated FBS, 100 mM of HEPES buffer, 55 μM of 2-ME, 100 U/mL of penicillin and 100 μg/mL of streptomycin. All cell culture works were performed in 37°C and 5% CO_2_ conditions.

### Tumor Challenge

LLC-OVA cells or EG7 cells (2 × 10^6^ cells/200 μL PBS) were subcutaneously (s.c.) injected into shaved back of mice. Tumor size was measured using calipers and determined by following formula: tumor volume (cm^3^) = (long diameter) × (short diameter)^2^ × 0.4. Antioxidants were intraperitoneally (i.p.) injected into mice: 500 μg of EGT (2.18 μmol) or 350 μg of NAC (2.14 μmol) was administrated. Cancer vaccination (15 nmol of Pam2CSK4 and 100 μg of OVA protein) was s.c. administrated into mice. For CD8^+^ T cell depletion, monoclonal antibody (Ab) against CD8β (clone: H35.17-2) was used in the form of ascites, which was i.p. injected into mice 1 day before vaccination. Ascites contains 10–15 mg/mL of IgG generally (25), and administration of 15 μL/head ascites was enough to deplete splenic CD8^+^ CD3^+^ cells according to the titration test.

### Flow Cytometry

Single-cell suspensions isolated from tissues were stained with fluorescence-labeled Abs after blockade with an anti-CD16/32 Ab. For T cell cytokine detection, samples were prepared by culturing total tumor cells for 6 h in 96 well U-bottom plate in the presence of 50 nM of SL8 peptide. To stop secretion, 10 μg/mL of Brefeldin A (Sigma-Aldrich) was added to the medium 1 h after peptide stimulation. Then, cells were fixed and permeabilized using BD Cytofix/Cytoperm Kit (BD Biosciences, CA, USA) and reacted with fluorescence-labeled Abs in the presence of 2% rat serum. Cells were fixed and permeabilized for detection of induced nitric oxide synthase (iNOS) or nitrotyrosine. Analysis was performed using FACS Aria II (BD Biosciences) and FlowJo software (Tree Star, CA, USA). Unless otherwise noted, all cell populations were explored on living cells, which was judged by 7-aminoactinomycin D (7AAD).

### Determination of Cell Viability or Proliferation

WST-1 assay reagent (Dojindo, Kumamoto, Japan) was used according to manufacturer's protocol. Cells were cultured in clear 96-well plate and A_450nm_ was determined after 2 h reaction with WST-1 reagent. The background values of assay medium were subtracted from determined values (EGT didn't affect the background value). LLC-OVA cells (5 × 10^3^ cells/well) or magnetically sorted F4/80^+^ cells (1 × 10^4^ cells/well) were applied to this assay.

### Cytometric Beads Assay (CBA)

Concentration of TNF-α, IFN-γ in tumor was determined by CBA (BD Biosciences). To prepare tumor lysates, tumor pieces about 15 mg were homogenized with CelLytic MT Mammalian Tissue Lysis/Extraction Reagent (Sigma-Aldrich) supplemented with Complete Protease Inhibitor Mixture (Roche). The lysis volume was unified as 10 μL reagent/1 mg tissue.

### Quantitative Reverse Transcription PCR (RT-qPCR)

Total RNA was isolated using TRIzol Reagent (Thermo Fisher Scientific). For tumor tissue sampling, small pieces were cut and collected into TRIzol reagent. RNA of culture cells was collected from 5 × 10^5^ cells. After recombinant DNase I (RNase-free, purchased from Takara, Tokyo, Japan) treatment, Reverse transcription was performed using High-Capacity cDNA Reverse Transcription Kit (Thermo Fisher Scientific). Power SYBR Green PCR Master Mix High-Capacity cDNA Reverse Transcription Kit (Thermo Fisher Scientific) was used for quantitative PCR assay and detection was conducted by StepOne Real-Time PCR System (Thermo Fisher Scientific). Gene expression levels normalized to GAPDH expression were depicted in the figures. Data were analyzed by the ΔΔCt method. Primer pairs are listed in Supplementary Table 2.

### *In vitro* CTL Activation and Co-culture With TAMs

CD8^+^ splenocytes (5 × 10^4^ cells) were isolated from tumor-free mice using CD8-microbeads, and then, cultured with or without 5 × 10^4^ intratumoral F4/80^+^ cells (magnetically sorted from tumor) in 96 well U bottom plate. F4/80^+^ cell cells were sorted after labeling with biotin-conjugated each Ab and streptavidin-microbeads. All fractions after sorting were purified in magnetic column again and used for studies. F4/80^+^ cells were incubated with or without 10 mM of EGT, 10 mM of HER, or 10 mM of NAC for 24 h before coculture with CD8^+^ splenocytes. T cell activation was achieved by stimulation with 0.25 μg/mL of anti-CD28 Ab and 0.1 μg/mL of anti-CD3 Ab as previously reported (26).

### Statistical Analysis

*P*-values were calculated by student's *t*-test or one-way analysis of variance (ANOVA) with Bonferroni's test in the case of two groups- or multiple- comparison, respectively. Calculations were performed using Microsoft excel (WA, USA) or GraphPad Prism 4 (CA, USA). Error bar represents the SEM between samples. Dot plot shows the parameter of each mouse, and the bar means average value.

## Results

### EGT Enhances CTL-Dependent Cancer Vaccine Therapeutic Efficacy Using the TLR2/6 Ligand

**Figure 1:**
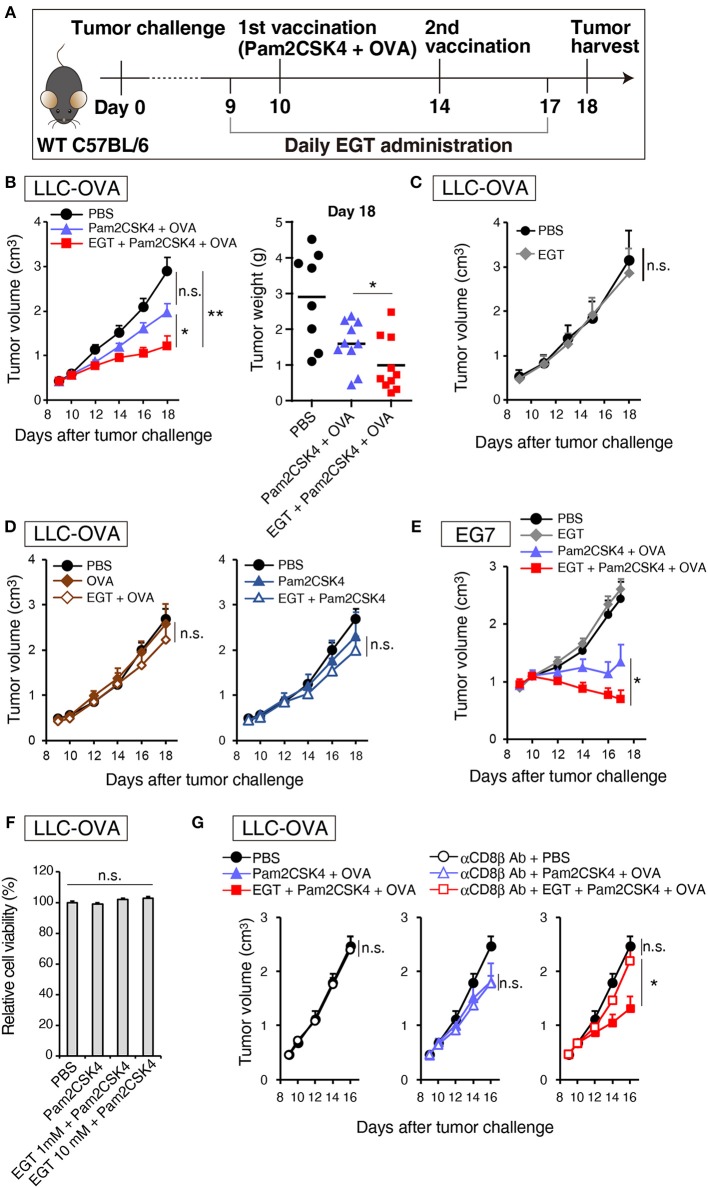
EGT augments tumor-growth retardation induced by cancer vaccine using TLR2/6 ligand. **(A)** Protocol for this study. **(B)** WT B6 mice were challenged with LLC-OVA cells (Day 0). Daily i.p. administration of 500 μg EGT was started on day 9 and vaccination (s.c. injection of 15 nmol Pam2CSK4 and 100 μg OVA protein) was performed on day 10 and 14. At day 18, tumors were harvested, and the weight was measured in addition to tumor volume. Data were pooled from two independent experiments with similar results. *n* = 9–10 mice per group. **(C)** Tumor growth in sole treatment of EGT. Schedule of EGT administration is shown in **(A)** Mice were challenged with LLC-OVA cells and i.p. treated with EGT alone or PBS control. At timed intervals, tumor growth was measured. *n* = 4 mice per group. **(D)** Mice were challenged with LLC-OVA cells and s.c. treated with OVA, EGT + OVA, Pam2CSK4 or EGT + Pam2CSK4. PBS was used as a control. Tumor growth was chased every other day. *n* = 4 mice per group. **(E)** Mice were challenged with EG7 tumor cells and treated with PBS, EGT, Pam2CSK4 + OVA, EGT + Pam2CSK4 + OVA as in **(A)**. Tumor growth were measured. *n* = 4–5 mice per group. **(F)** LLC-OVA cells were cultured with 1 or 10 mM EGT for 24 h, and then treated with 50 nM Pam2CSK4. Cell viability was assessed by WST-1 reagent 48 h after Pam2CSK4 treatment. *n* = 3–4. **(G)** Anti-CD8β antibody was i.p. injected into LLC-OVA-bearing mice on day 9 and 13 and the treatment described in **(A)** was performed. *n* = 4–5 mice per group. *P < 0.05, **P < 0.01. n.s., not significant.

Pam2CSK4, a ligand of the TLR2/6 heterodimer, was used as an adjuvant for a cancer vaccine in combination with TAA (OVA in this case). Figure 1A is a protocol for evaluation of EGT function on vaccine therapy in mouse tumor models. In the LLC-OVA model, vaccination with Pam2CSK4 and OVA protein barely showed significant tumor growth retardation (Figure 1B). However, in combination with EGT, Pam2CSK4 + OVA significantly suppressed tumor growth (Figure 1B). EGT *per se* neither had no tumor-suppressive function (Figure 1C) nor enhanced tumor growth retardation with either OVA or Pam2CSK4 alone (Figure 1D). EGT also augmented tumor suppression by Pam2CSK4 + OVA in another OVA-positive EG7 tumor model, although EG7 tumor was more susceptible to Pam2CSK4 + OVA than LLC-OVA tumor (even without EGT) (Figure 1E). Thus, EGT greatly contributes to tumor regression only in the presence of antigen and adjuvant. WST-1 proliferation assays showed that EGT did not have direct cytotoxicity to LLC-OVA cells even in the presence of Pam2CSK4 (Figure 1F). CD8^+^ T cell depletion studies (Figure 1G) suggested that tumor regression occurring with combination treatment of Pam2CSK4 + OVA + EGT was largely dependent on CD8β^+^ T cells.

### EGT Increases Intratumoral CTL Functionality

**Figure 2:**
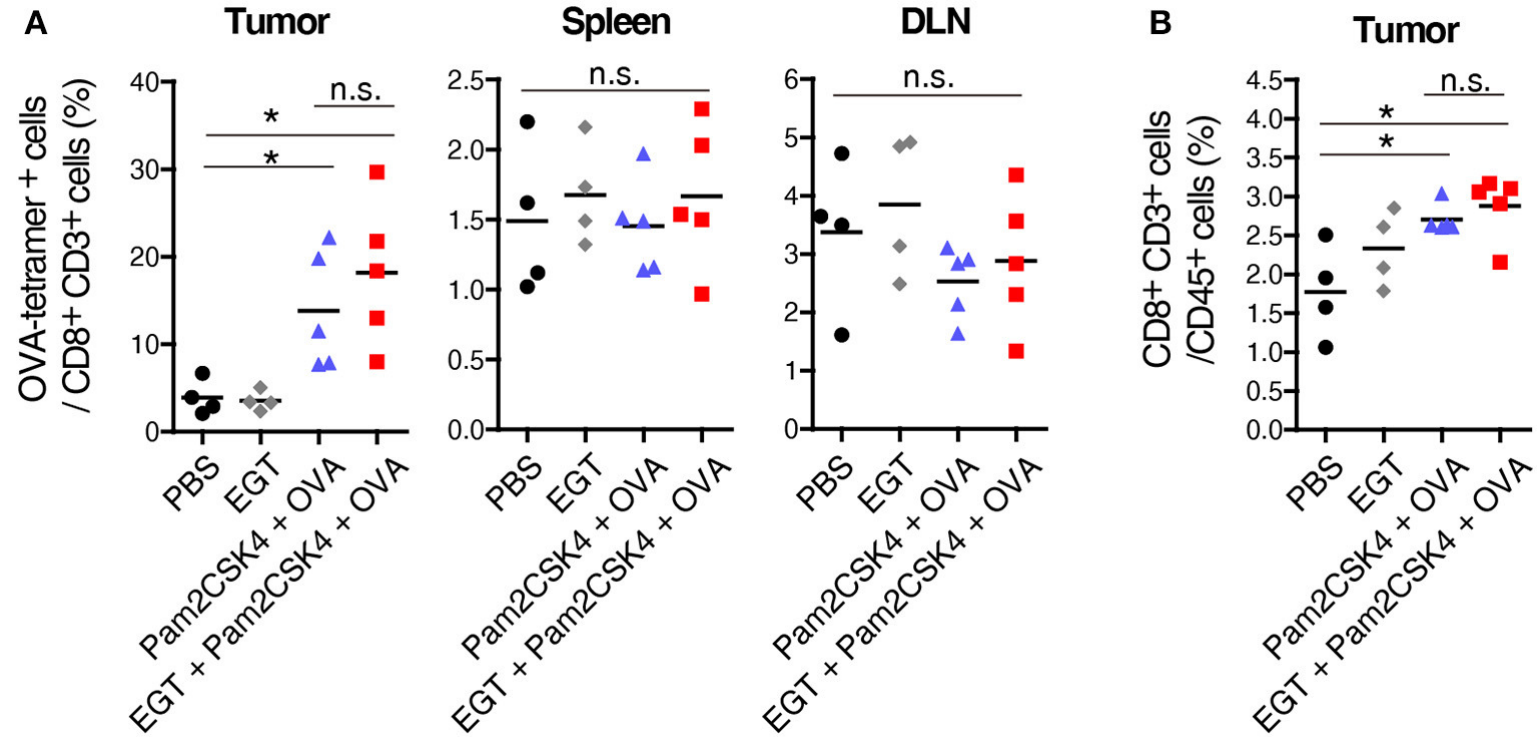
EGT does not increase priming and tumor-infiltration of CTLs. LLC-OVA-implanted WT B6 mice were treated as per Figure 1A. At day 18, tumor, spleen and draining lymph nodes (DLN: inguinal LN) were harvested. Then, population of OVA-tetramer positive cells **(A)** and total CD8^+^ CD3^+^ cells in tumor **(B)** were counted by flowcytometry. *n* = 4–5 mice per group. *P < 0.05. n.s., not significant.

Pam2CSK4 + OVA but not EGT alone increased the number of OVA-specific CD8^+^ T cells in tumor environment (Figure 2A). However, CD8^+^ T cells was not elevated in the spleen or draining lymph nodes (DLN) in the same mice (Figure 2A), which was judged with authentic SL8 (OVA)-tetramer assay. Pam2CSK4 + OVA administration increased tumor-infiltrating CD8^+^ T cells but addition of EGT did not affect CD8^+^ T cell infiltration (Figure 2B). Generally, cancer vaccine induces cross presentation of DCs, resulting in expansion of antigen-specific CTLs in lymphoid organs and tumors. Hence, Pam2CSK4 + OVA worked as a cancer vaccine, but EGT did not increase cross presentation or priming *in vivo*. EGT alone did not enhance tumor infiltration of CTLs (Figure 2). Thus, EGT may function in intratumoral activation of CTLs, the phase after CTL infiltration.

**Figure 3:**
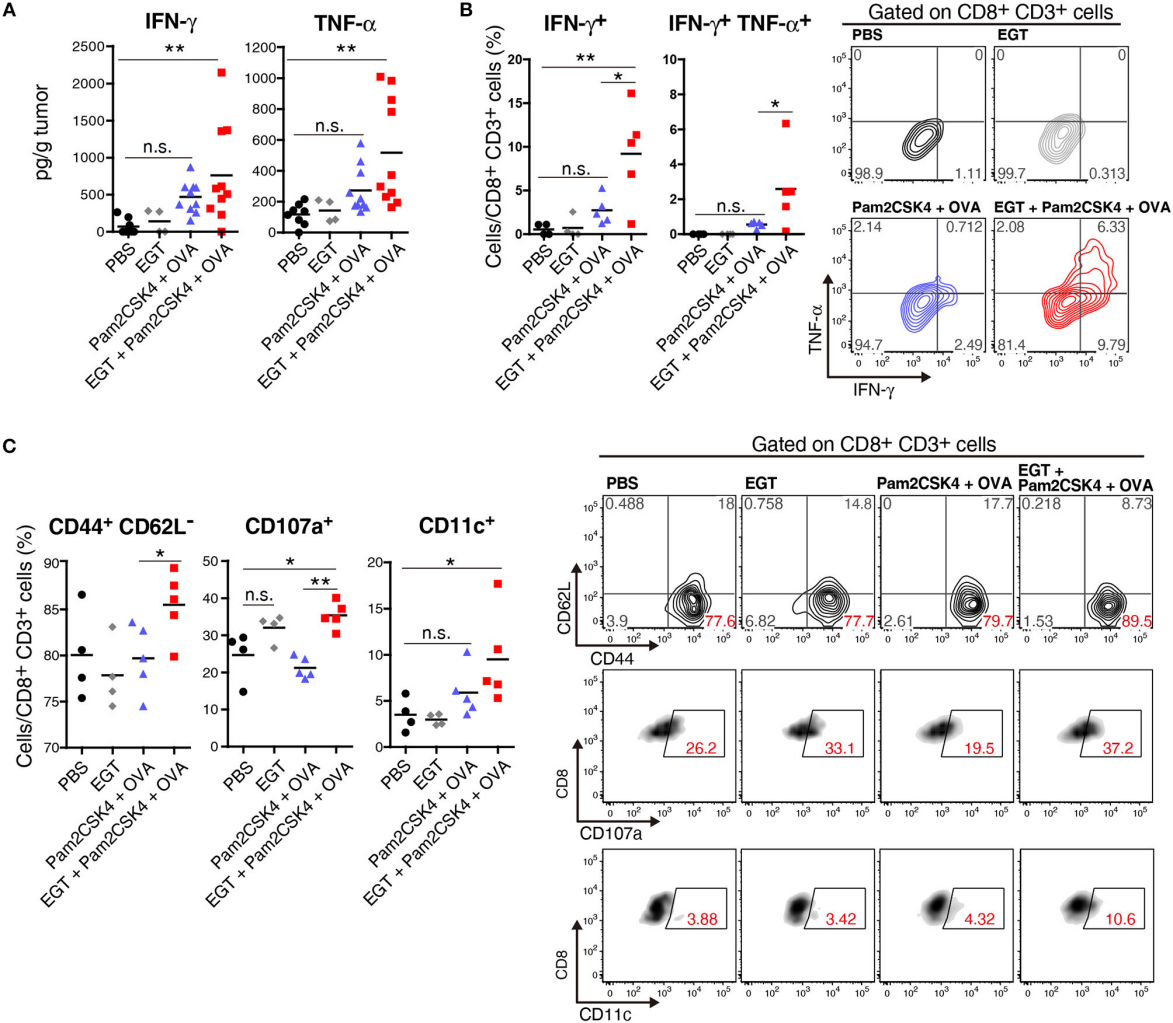
EGT improves CTL functionality in tumor in combination with TLR2/6 ligand. **(A–C)** LLC-OVA-implanted WT B6 mice were treated as per Figure 1A and tumors were harvested at day 18. **(A)** Concentration of IFN-γ and TNF-α in tumor was determined by cytometric beads assay (CBA). Data were pooled from two independent experiments resulted in similar profiles. *n* = 4–10 mice per group. **(B)** Total tumor cells were re-stimulated with 50 nM of SL8 peptide for 6 h. Brefeldin A was supplied during last 5 h of culture. Then, intracellular expression levels of IFN-γ and TNF-α in CD8^+^ CD3^+^ cells were detected by flowcytometry. Representative data are shown as FACS plot. *n* = 4–5 mice per group. **(C)** Cell surface expression of indicated proteins on CD8^+^ CD3^+^ cells were analyzed by flowcytometry. *n* = 4–5 mice per group. *P < 0.05, **P < 0.01. n.s., not significant.

CTL functionality can be assessed by cytokine production (27). We determined the intratumor content of IFN-γ and TNF-α which are representative cytokines released from activated CTLs. Both IFN-γ and TNF-α subtly increased in the Pam2CSK4 + OVA group and more clearly increased in the EGT + Pam2CSK4 + OVA group compared to the PBS group or EGT single-treated group (Figure 3A). To examine if the cytokines were produced by CTLs, we detected IFN-γ^+^ and TNF-α^+^ cells using flow cytometry after stimulation with SL8 peptide. Consistent with the cytokine results, Pam2CSK4 + OVA minimally increased IFN-γ/TNF-α-positive CD8^+^ CD3^+^ cells compared to the PBS group, and the combination of EGT led to significant increases of IFN-γ/TNF-α in the CD8^+^ CD3^+^ cells (Figure 3B).

The activation state of CTLs was further evaluated by cell surface markers. We quantified the fraction of CD44^+^ CD62L^−^ cells (to test an effector/memory state) (27), CD107a^+^ cells (to test a secretion state of cytotoxic molecules) (28) and CD11c^+^ cells (a highly tumoricidal subset) (29) per CD8^+^ CD3^+^ cells. Flow cytometric analysis demonstrated that these activation fractions were greatly increased in the EGT + Pam2CSK4 + OVA group compared to the Pam2CSK4 + OVA group (Figure 3C). In this context, we evaluated the levels of molecules possibly involved in CTLs exhaustion (27). PD-1, LAG-3, and Tim-3 are representative molecules reflecting CTL exhaustion (27). Yet, no decrease in PD-1, LAG-3, and Tim-3 was observed in the EGT + Pam2CSK4 + OVA group compared to the Pam2CSK4 + OVA group (Supplementary Figure 1).

EGT had no function on αCD3/CD28 Ab-induced direct activation of CTLs (Supplementary Figure 2). Thus, EGT acts on tumor microenvironment surrounding CTLs. Because EGT can modulate macrophage TLR responses (16), we next analyzed whether TAMs suppress CTL function.

### EGT Decreases TAM Proliferation Induced by TLR2/6 Vaccination

**Figure 4:**
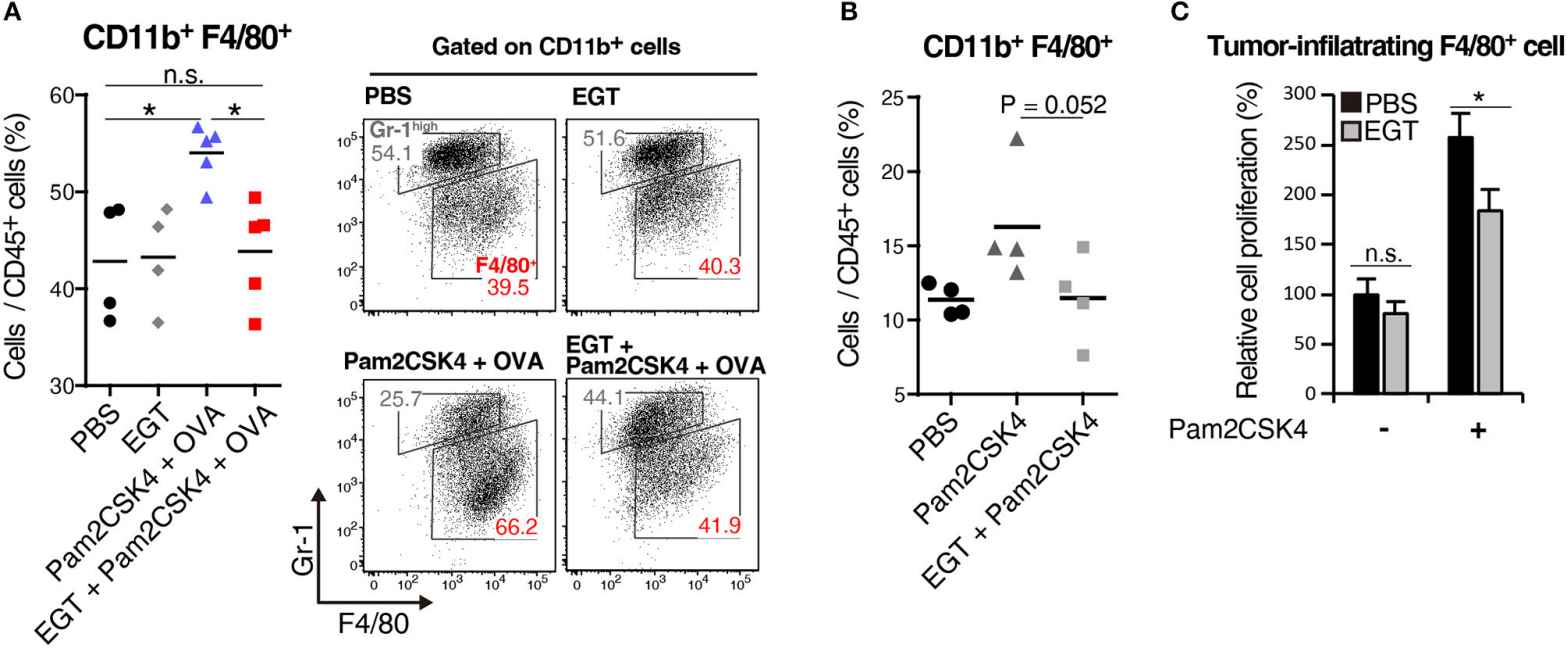
EGT cancels TAM proliferation induced by TLR2/6 ligand. **(A,B)** LLC-OVA-implanted WT B6 mice were treated with PBS, EGT, and/or Pam2CSK4 + OVA, as per Figure 1A and tumors were harvested at day 18. In **(B)**, mice were injected with Pam2CSK4 without OVA. Tumor-infiltrating cells were analyzed by flow cytometry to count the number of CD11b^+^ Gr-1^−/low^ F4/80^+^ cells. Representative data are shown as FACS plot. *n* = 4–5 mice per group. **(C)** Sorted tumor-infiltrating F4/80^+^ cells were cultured with or without EGT for 24 h, and then, cells were stimulated with 50 nM of Pam2CSK4. WST-1 assay was performed 72 h after Pam2CSK4 treatment. *n* = 3. *P < 0.05. n.s., not significant.

We elucidated the amount of Gr-1^−/low^ F4/80^+^ TAMs in tumor by flow cytometry. Pam2CSK4 + OVA increased F4/80^+^ TAMs, and EGT reversed this population shift (Figure 4A). Pam2CSK4 without OVA also induced a similar response (Figure 4B). To evaluate the mechanism of this change, we determined intratumoral expression of growth factors and chemokines (*Ccl2, Cxcl12, Csf1*, and *Il34*) related to proliferation and infiltration of TAMs (30, 31). No change was observed in TAM-related factors (Supplementary Figure 3). TAMs were harvested from tumors and cultured with or without Pam2CSK4 and/or EGT. TAM proliferation induced by Pam2CSK4 was suppressed by EGT (Figure 4C). Thus, EGT interferes with TAM growth/survival signaling in response to TLR2/6 stimulation.

### EGT Reduces TAM Suppressive Phenotype Under TLR2/6 Stimulation Dependent on Thiol/Thione Structure

**Figure 5:**
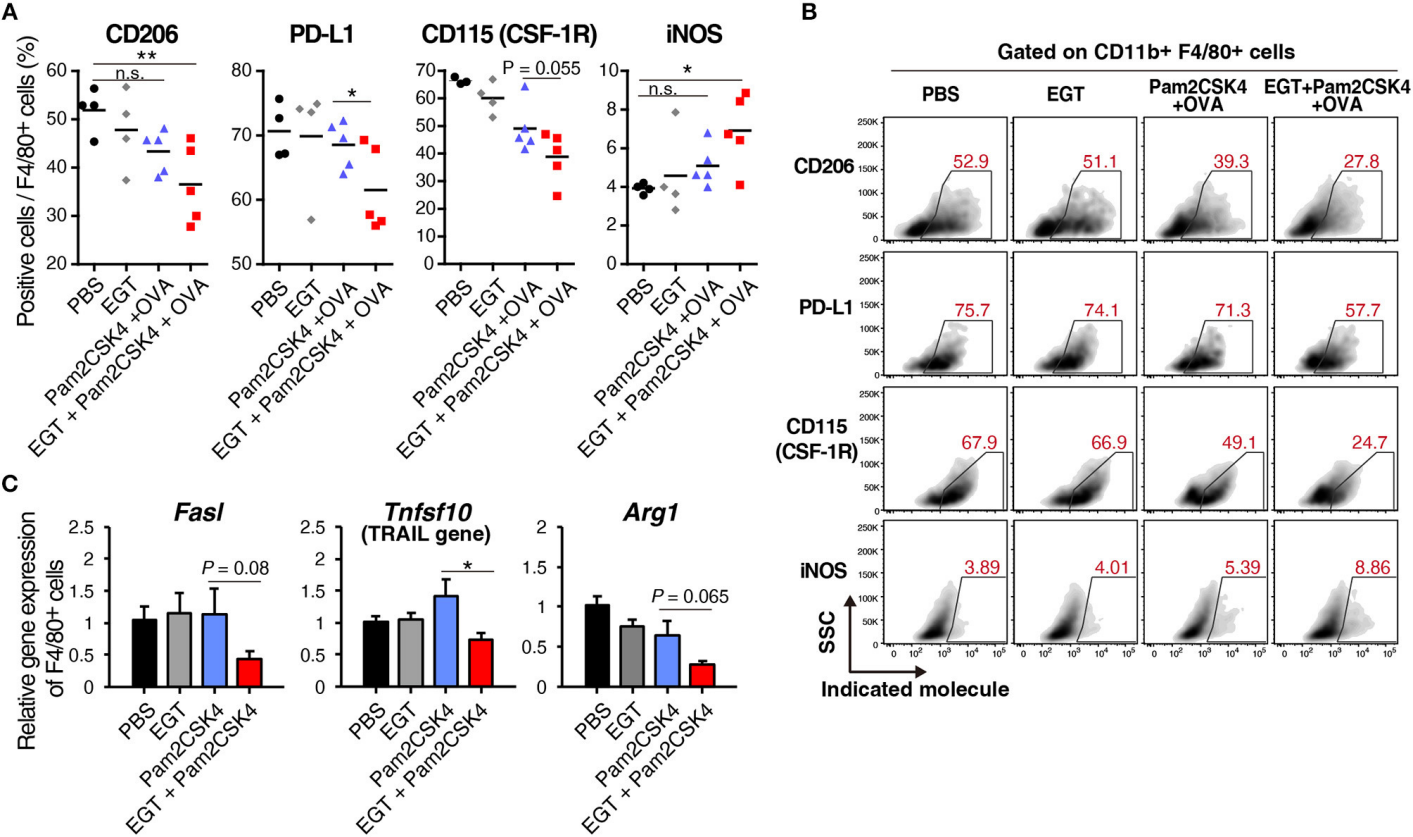
EGT converts TAMs into less-suppressive phenotype under TLR2/6 stimulation. **(A,B)** LLC-OVA-implanted WT B6 mice were treated as shown in Figure 1A and tumors were harvested at day 18. *n* = 4–5 mice per group. **(A)** Expression of indicated proteins of CD11b^+^ Gr-1^−/low^ F4/80^+^ cells were analyzed by flowcytometry. **(B)** Representative FACS plots of the data in Figure 6A. **(B)** Sorted tumor-infiltrating F4/80^+^ cells were treated with or without 10 mM of EGT for 24 h, and then, stimulated with 50 nM of Pam2CSK4. Total RNA was collected 4 h after Pam2CSK4 stimulation. *n* = 3. *P < 0.05, **P < 0.01. n.s., not significant.

EGT altered molecular expression of TAMs under Pam2CSK4 + OVA treatment. CD206, a marker of immunosuppressive macrophages, and PD-L1, a representative molecule that induces CTL exhaustion, were downregulated in the EGT + Pam2CSK4 + OVA group (Figures 5A,B). CD115 (CSF-1R) expression was lowest in the EGT + Pam2CSK4 + OVA group (Figures 5A,B). CD115 gives TAMs growth stimulation and immunosuppressive properties (32). Thus, combining EGT with TLR2-containing cancer vaccine will relieve CTLs from dysfunction induced by TAMs.

Induced nitric oxide synthase (iNOS), a marker of inflammatory macrophages, was upregulated in the EGT + Pam2CSK4 + OVA group (Figures 5A,B). Expression of iNOS is a prognosis marker for long survival of cancer patients (33), but some reports indicate that NO, the product of an iNOS reaction, suppresses CTLs by nitration of T cell molecules (13, 34). To investigate whether nitration occurs in T cells in response to EGT and Pam2CSK4, we measured nitrotyrosine levels of CTLs (34). CTL nitration was not enhanced in the EGT + Pam2CSK4 + OVA group (Supplementary Figure 4), which suggests that increased iNOS expression was not harmful in T cells in this model.

We further analyzed RNA expression levels of TAM molecules, which impedes CTL function. EGT decreased expression of the *Fasl, Tnfsf10*, and *Arg1* genes under Pam2CSK4 stimulation (Figure 5C). FAS ligand and TRAIL expressed by TAMs are involved in death of CTLs. Arginase-1 causes CTL dysfunction by nutritious depletion (3, 4). Our results indicate that EGT improves the tumor immune environment formed in response to TLR2/6 ligand by modulating cellular signaling and functions in TAM.

### EGT Reverses TAM-Induced CTL Inactivation Under TLR2/6 Stimulation

**Figure 6:**
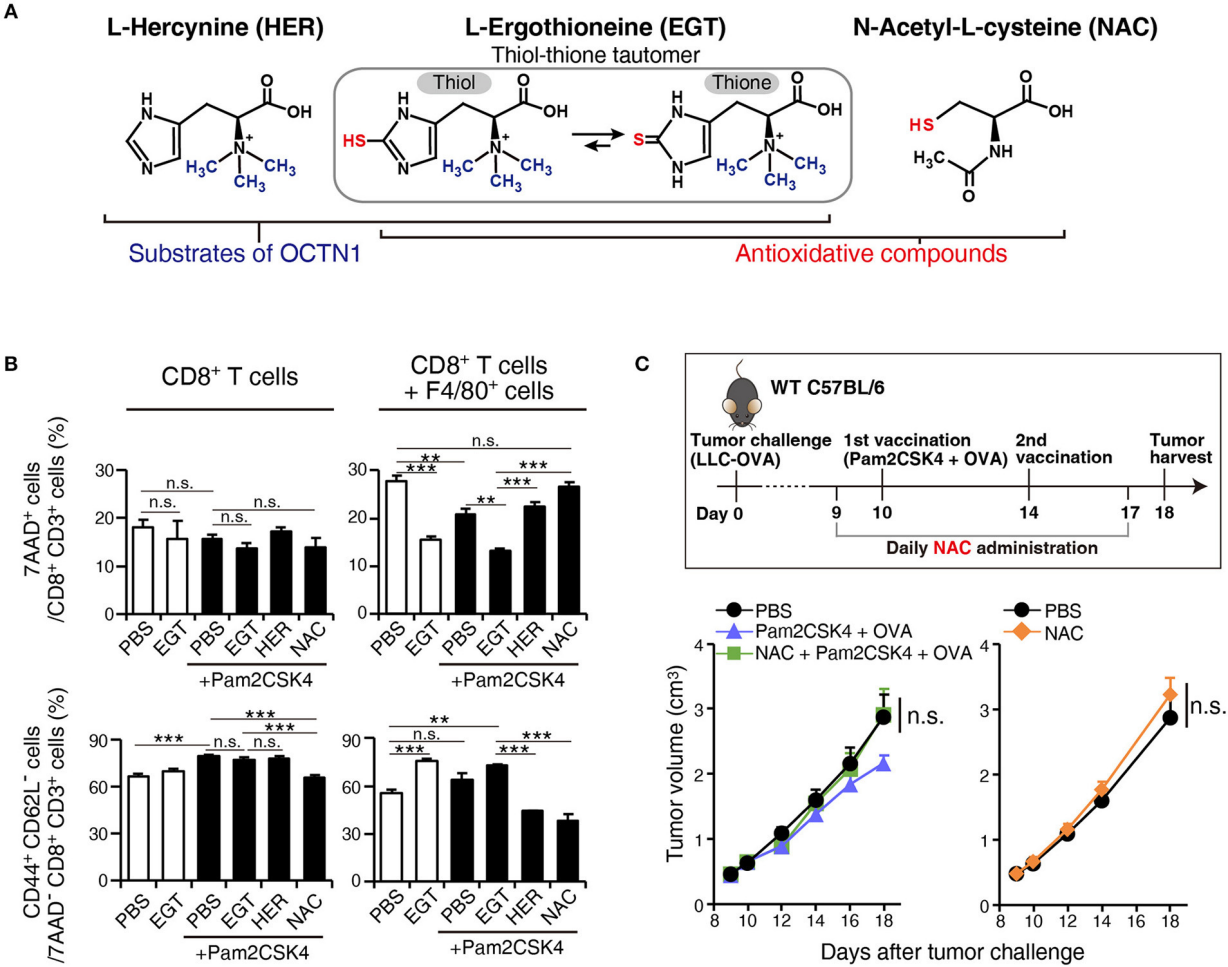
EGT relieves TAM-induced decrease of CTL viability/activity depending upon thiol-thione residue. **(A)** The compound structure of HER, EGT or NAC. **(B)** F4/80^+^ cells were cultured with/without 10 mM of EGT, HER or NAC for 24 h. Then, CD8^+^ splenocytes were mixed in 1:1 ratio. Promptly, the cells were stimulated with 0.1 μg/mL of αCD3 Ab and 0.25 μg/mL of αCD28 Ab. After 60 h co-culture and Ab stimulation, non-adherent cells were collected. The ratios of the 7AAD^+^ cell and CD44^+^ CD62L^−^ cell populations in CD8^+^ CD3^+^ T cells were determined using flow cytometry. Cells in all conditions were obtained and analyzed at the same time. *n* = 3. **(C)** WT B6 mice were challenged with LLC-OVA cells (Day 0). Daily i.p. administration of 350 μg NAC (an equimolecular amount of 500 μg EGT) was started on day 9 and vaccination (s.c. injection of 15 nmol Pam2CSK4 and 100 μg OVA protein) was performed on day 10 and 14. *n* = 4–5 mice per group. **P < 0.01,***P < 0.001. n.s., not significant.

To ensure that EGT combined with TLR2/6 ligand eliminated CTL suppression by TAMs, we constructed a co-culture assay system of tumor-infiltrating F4/80^+^ macrophages and CD8^+^ splenocytes to test the reversal of CTL suppression by EGT treatment and TLR2/6 ligand. The importance of the anti-oxidative thiol-thione residue of EGT was also evaluated by comparison to responses to L-hercynine (HER) which is a thiol-thione-free form of EGT. Another anti-oxidant, N-acetyl-L-cysteine, was also compared with EGT. The compound structures are in Figure 6A.

F4/80^+^ cells were treated with EGT, HER, or NAC for 24 h before co-culture. CD8^+^ splenocytes were added to wells and stimulated with αCD3 Ab + αCD28 Ab. To determine if CTL functions were suppressed by co-culture with TAMs, we detected 7AAD^+^ dead cells and CD44^+^ CD62L^−^ effector/memory cells.

Co-culture of CD8^+^ splenocytes with intratumoral F4/80^+^ cells increased 7AAD^+^ dead CTLs and decreased CD44^+^ CD62L^−^ effector/memory CTLs (Figure 6B). In alignment with the *in vivo* experiments, EGT increased effector/memory phenotypes of CTLs, accompanied by a decreased 7AAD^+^ fraction. This result occurred with or without TLR2/6 stimulation, which might be the response to mediators from activated CTLs that stimulate macrophages. HER (with no SH residue) did not improve the viability or activity of CTLs in co-culture with TAMs (Figure 6B). Hence, the thiol-thione of EGT is essential to overcome the immune suppression of TAMs. Moreover, the anti-oxidant NAC failed to recover CTL functionality with co-culture with TAMs. NAC also failed to improve the therapeutic efficacy of vaccination by Pam2CSK4 + OVA *in vivo* (Figure 6C). This result highlights mechanistic differences between EGT and NAC on oxidation-reduction (redox) signaling to control suppressive function of TAMs.

## Discussion

**Figure 7:**
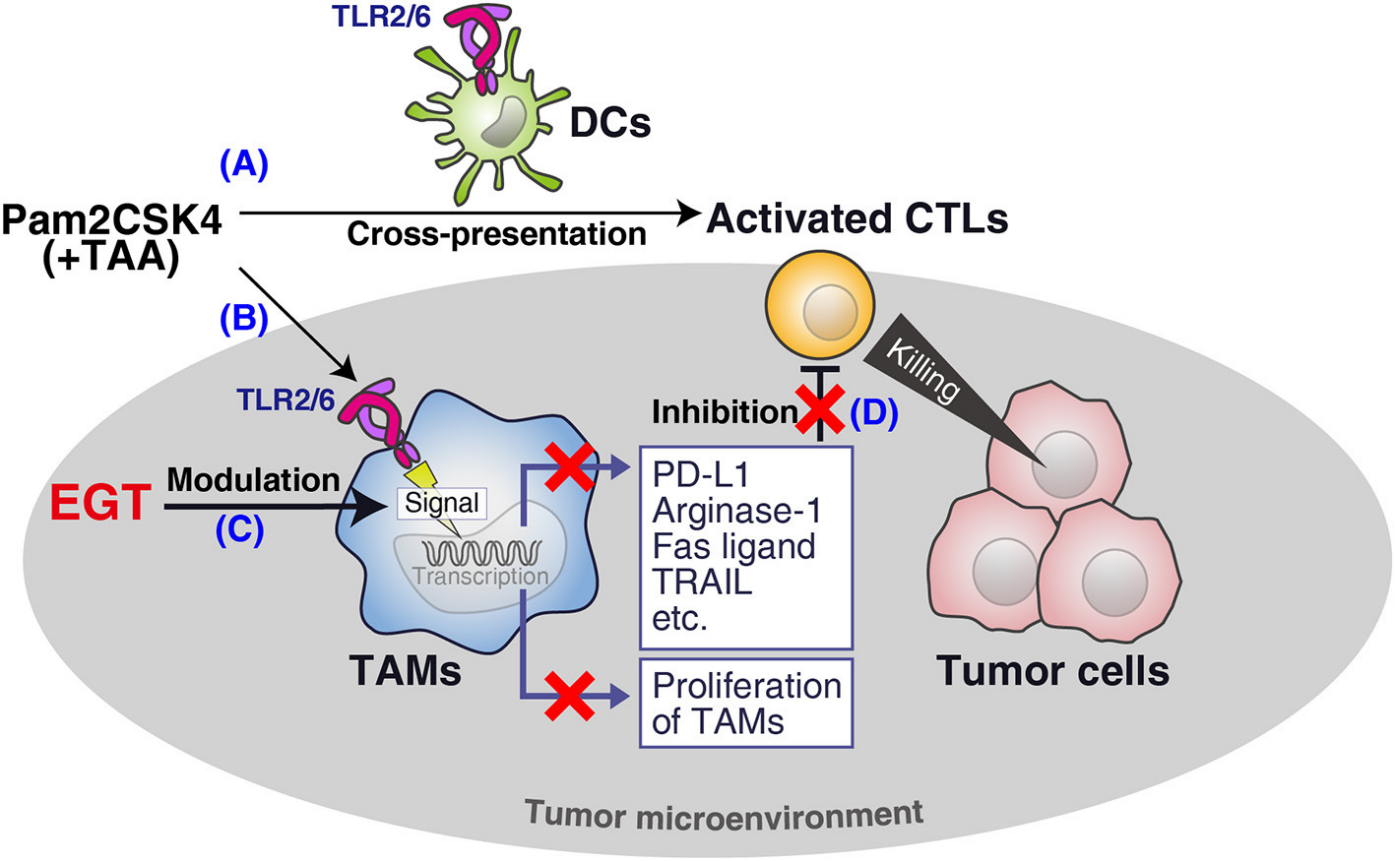
Summary of this study. **(A)** Pam2CSK4 and TAA induce DC maturation and CTL priming, which induces tumor cell death. **(B)** However, TLR2 stimulation increases immunosuppressive TAM proliferation. **(C)** EGT cancels the proliferation and further renders TAM less immuno-suppressive in combination with TLR2/6 ligand. **(D)** The EGT function in tumor leads to improved functionality of intratumoral CTLs and enhances therapeutic efficacy of the vaccination.

We demonstrated that EGT was an anti-oxidant that improved cancer immunotherapy using TLR2/6 ligand *in vivo* (Figure 7). EGT ameliorated the functionality of intratumor CTLs and had little effect on DC potential or CTL activation in TLR2-mediated priming. We showed that stromal macrophages around CTLs in tumors were major targets for EGT. EGT combined with TLR2 ligand decreased the number of TAMs and caused iNOS upregulation and CD206 downregulation, indicating the conversion of TAMs into an inflammatory phenotype. This TAM polarization triggered microenvironmental alterations that made intratumoral CTLs effective in concordance with previous reports (33, 35, 36). As a result, EGT/adjuvant vaccine therapy efficiently controls tumor growth in murine models.

CD115 (CSF-1R) and PD-L1 are involved in the immunosuppressive function of TAMs (32, 37), and are downregulated by a combination of TLR2 vaccine and EGT. *Ex vivo* analysis showed that TAMs decreased the expression of FASL and TRAIL in response to EGT and TLR2 stimulation, which are related to CTL apoptosis and nutritional depletion (due to arginase-1) (3), and sustained the survival and activity of CTLs. EGT improved the viability and effector/memory activities of CTLs in co-culture assays with TAMs. Hence, EGT reorganized the microenvironment by phenotypic alteration of TAMs to switch from an immune-suppressive to an immune-reactive environment.

While thiol/thione-compound EGT attenuates TAM suppressive function, HER, a non-thiol/thione EGT, does not rescue CTLs in co-culture assay with TAMs (Figure 6B). HER had enough modulatory function on cytokine production as same as EGT (Supplementary Figure 5). The results imply that anti-oxidative activity was critical to TAM modulation by EGT except for cytokine regulation.

NAC, another antioxidant, also failed to reverse CTL dysfunction in co-culture assays. *In vivo* experiments also demonstrated that NAC did not improve adjuvant immunotherapy using TLR2 ligand. This result demonstrates the presence of EGT-specific redox signal regulation. The physiological difference between EGT and NAC may explain EGT-specific redox control. The strength of anti-oxidants, especially those containing thiol, is represented by the redox potential of the thiol-disulfide couple. Generally, the standard redox potential of naturally occurring thiols is between −230 and −320 mV. More negative values indicate more potent anti-oxidative properties (17). Glutathione, an active metabolite of NAC, has a redox potential of about −250 mV (38). However, the thiol-thione of EGT has a redox potential of −60 mV (17). If strong reduction of redox signaling by NAC is a disadvantage for modulating the tumor environment, mild anti-oxidation by EGT may achieve the unique responses to improve the environment.

EGT and NAC are different in the cell types they enter. *In vivo* NAC treatment suppressed ROS production in both TAMs and CD45^−^ tumor cells, but EGT failed to decrease ROS in CD45^−^ tumor cells despite the scavenging action of TAMs (Supplementary Figure 6). This response is helpful for cancer treatment. Because the therapeutic effect of radiotherapy and some chemotherapies depend on ROS production and subsequent cell death (39), EGT might not disturb pre-existing cancer therapy. The established safety of EGT (40–42) further endorses the utility of EGT for cancer patients.

In contrast to the tumor-suppressive functions, TLR2 ligands enhance tumor progression by modulating macrophage function (12–15). Tumor-progressive responses are limiting factors of cancer vaccines using TLR2 ligands. In TAM-rich tumor such as LLC-OVA, the percentage of F4/80^+^ macrophages is higher than in TAM-poor tumor as in EG7 (23). This may be a reason why LLC-OVA tumor was less susceptible to Pam2CSK4 + OVA than EG7 tumor (Figures 1B,E). TAM expansion occurs in response to TLR2 ligand in tumor through subcutaneous local injection (Figure 4A). Because Pam2CSK4 is a small molecule (molecular weight: 1271.83), it is possibly delivered to whole body via the circulation. EGT is also a small molecule and delivered to macrophages in tumor (Supplementary Figure 6). Thus, EGT modulates the function of TLR2 ligand in tumor macrophages, which verifies TLR2's beneficial response for cancer vaccine therapy.

Our data demonstrate that EGT targets tumor macrophages to potentiate TLR2 ligand action against tumor progression. EGT may enable TLR2 ligand to contribute to the development of cancer immunotherapy.

## Author Contributions

SY, HS, MM, and TS contributed to the conception and design, contributed to the analysis and the interpretation of data, and contributed to writing the manuscript. SY contributed to the acquisition of data. SY and HS contributed to the development of methodology. MM, MK, and TS contributed to the project administration and supervision.

### Conflict of Interest Statement

The authors declare that the research was conducted in the absence of any commercial or financial relationships that could be construed as a potential conflict of interest.
