# Supplementary material for: Anti-oxidative Amino Acid L-ergothioneine Modulates the Tumor Microenvironment to Facilitate Adjuvant Vaccine Immunotherapy
Source: Front Immunol. 2019 Apr 4;10:671. doi: 10.3389/fimmu.2019.00671 (PMC6458301; doi:10.3389/fimmu.2019.00671)
Supplement: Supplementary file 1 [file Data_Sheet_1.PDF]

Supplementary Table 1. The list of antibodies and pigments used in this study.

Antibody or pigment	Clone	Dilution to use	Catalog number	Supplier
LEAF purified anti-mouse CD3ε Antibody	145-2C11	-	100314	BioLegend
LEAF purified anti-mouse CD28 Antibody	37.51	-	102111	BioLegend
Purified anti-mouse CD16/32 Antibody	93	×200	101302	BioLegend
Purified anti-mouse CD107a Antibody	1D4B	×200	121601	BioLegend
Anti-Nitrotyrosine (rabbit immunoaffinity purified IgG)	Polyclonal	×200	06-284	Merck
Biotin anti-mouse Ly-6G/Ly-6C (Gr-1) Antibody	RB6-8C5	×200	108403	BioLegend
Biotin anti-mouse F4/80 Antibody	BM8	×200	123105	BioLegend
Donkey anti-Rat IgG (H+L) Secondary Antibody, PE	polyclonal	×200	12-4822-82	eBioscience (Thermo Ficher Sciences)
F(ab')2-Donkey anti-Rabbit IgG (H+L) Secondary Antibody, PE	polyclonal	×200	12-4739-81	eBioscience (Thermo Ficher Sciences)
Streptavidin-PE/Cy7	-	×200	405206	BioLegend
FITC anti-mouse F4/80 Antibody	BM8	×200	123107	BioLegend
FITC anti-mouse IFN-γ Antibody	XMG1.2	×200	505806	BioLegend
PE anti-mouse CD8a Antibody	53-6.7	×200	100707	BioLegend
Anti-Mouse CD62L Antibody PE	MEL-14	×200	12-0621-81	eBioscience (Thermo Ficher Sciences)
Anti-Mouse CD115(c-fms) Antibody PE	AFS98	×200	12-1152-81	eBioscience (Thermo Ficher Sciences)
FITC anti-mouse CD206 (MMR) Antibody	C068C2	×200	141703	BioLegend
PE anti-mouse CD223 (LAG-3) Antibody	C9B7W	×200	125207	BioLegend
Anti-Mouse CD274 (B7-H1) Antibody PE	MIH5	×200	12-5982-81	eBioscience (Thermo Ficher Sciences)
PE anti-mouse CD279 (PD-1) Antibody	RMP1-30	×200	109103	BioLegend
PE anti-mouse F4/80 Antibody	BM8	×200	123109	BioLegend
Anti-Mouse NOS2 Antibody PE	CXNFT	×200	12-5920-80	eBioscience (Thermo Ficher Sciences)
T-select H-2Kb OVA Tetramer-SIINFEKL-PE	-	×50	TS-5001-1C	MBL
PE/Cy7 anti-mouse CD3 Antibody	17A2	×200	100219	BioLegend
PE/Cy7 anti-mouse/human CD11b Antibody	M1/70	×200	101215	BioLegend
PE/Cy7 anti-mouse CD366 (Tim-3) Antibody	B8.2C12	×200	134009	BioLegend
APC/Cy7 anti-mouse CD45 Antibody	30-F11	×200	103116	BioLegend
APC anti-mouse CD3ε Antibody	145-2C11	×200	100311	BioLegend
APC anti-mouse CD11c Antibody	N418	×200	117310	BioLegend
APC anti-mouse/human CD44 Antibody	IM7	×200	103012	BioLegend
APC anti-mouse Ly-6G/Ly-6C (Gr-1) Antibody	RB6-8C5	×200	108411	BioLegend
Alexa Fluor 700 anti-mouse CD3 Antibody	17A2	×200	100216	BioLegend
Alexa Fluor 700 anti-mouse CD8a Antibody	53-6.7	×200	100730	BioLegend
BD Via-Probe Cell Viability Solution	-	×50	555816	BD Bioscinences

Supplementary Table 2. The list of primer sequences for qPCR used in this study.

Gene	Forword priemer sequence (5'-3')	Reverse primer sequence (5'-3')
Arg1	GGAATCTGCATGGGCAACCTGTGT	AGGGTCTACGTCTCGCAAGCCA
Csf1	CCAAGGAGGTGTCAGAACACTGT	AAAGGCAATCTGGCATGAAGTC
Csf3	GAGCAGTTGTGTGCCACCTACA	AGCTGGCTTAGGCACTGTGTCT
Ccl2	AGGTGTCCCAAAGAAGCTGTAGTT	ACAGACCTCTCTCTTGAGCTTGGT
Cxcl1	TGAGCTGCGCTGTCAGTGCCT	AGAAGCCAGCGTTCACCAGA
Cxcl2	GTTAGCCTTGCCTTTGTTCAGTATC	GAGCTTGAGTGTGACGCCCCCAGG
Cxcl12	CAGAGCCAACGTCAAGCA	AGGTACTCTTGGATCCAC
Fasl	TTAAATGGGCCACACTCCTC	ACTCCGTGAGTTCACCAACC
Gapdh	GCCTGGAGAAACCTGCCA	CCCTCAGATGCCTGCTTCA
Ifnb	CCAGCTCCAAGAAAGGACGA	CGCCCTGTAGGTGAGGTTGAT
Il12a	TGTGTCTCCCAAGGTCAGC	ATGACCCTGGCCAAACTGAG
Il34	ACTCAGAGTGGCCAACATCACAAG	ATTGAGACTCACCAAGACCCACAG
Tgfb1	GCTGAACCAAGGAGACGGAAT	CAAGAGCAGTGAGCGCTGAA
Tnfsf10	CTTCACCAACGAGATGAAGCAG	TCCGTCTTTGAGAAGCAAGCTA

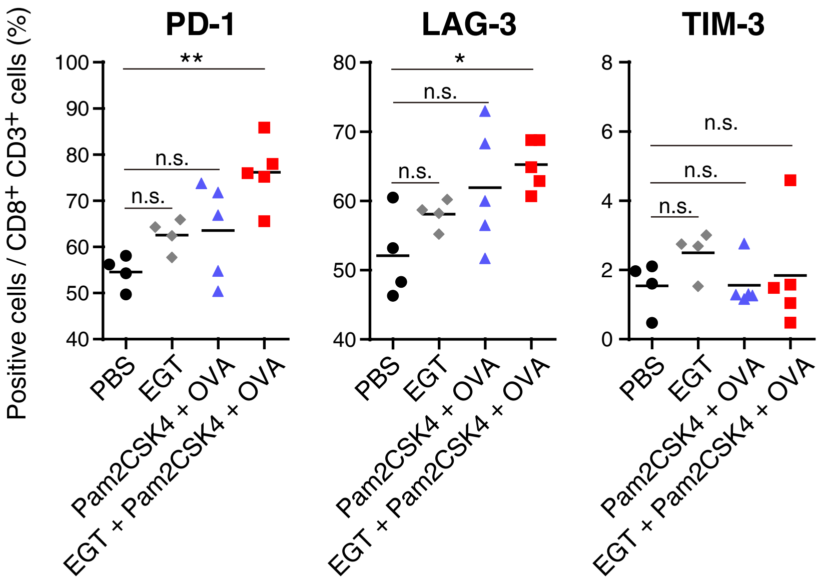

Supplementary Fig. 1. Expression analysis of exhaution markers on intratumoral CD8⁺ CD3⁺ cells. LLC-OVA-implanted WT B6 mice were treated as per Fig. 1A and tumors were harvested at day 18. Cell surface expression levels of indicated proteins on CD8+ CD3+ cells were analyzed by flow cytometry. n = 4-5 mice per group. *P < 0.05, **P < 0.01. n.s., not significant.

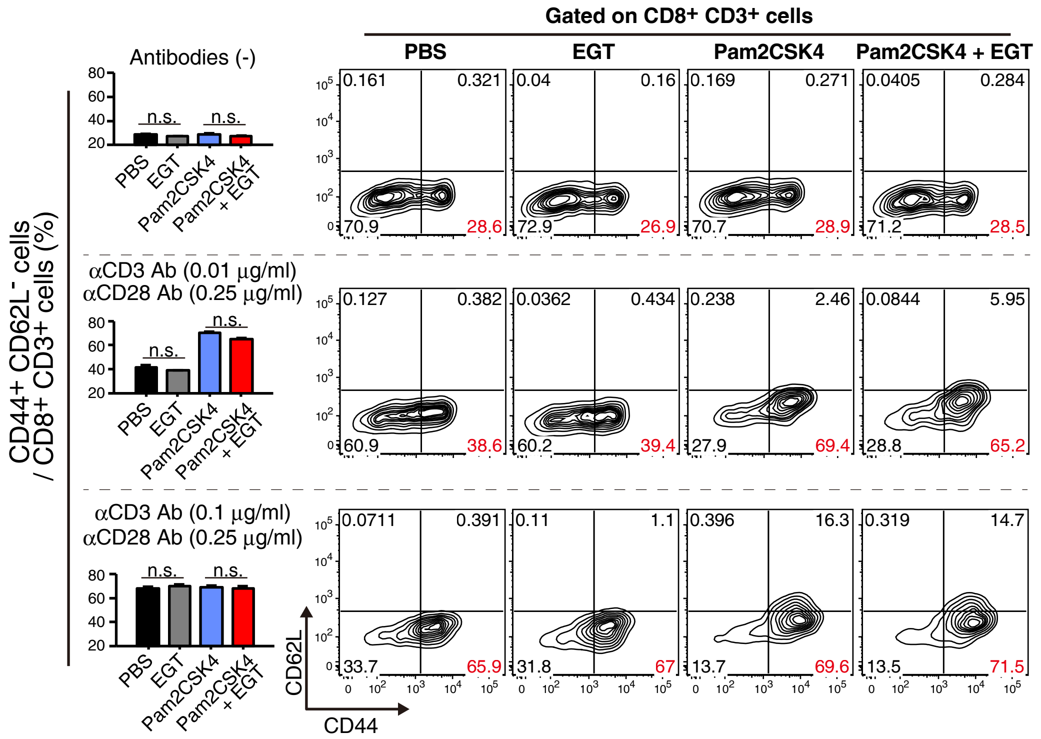

Supplementary Fig. 2. EGT does not induce direct activation of CTLs. $CD8^+$ splenocytes (1×10^5 cells/well in U-bottom plate) isolated from tumor-free mice were incubated with or without EGT for 24 h, and then, stimulation was performed with indicated concentration of αCD3 and αCD28 Abs. $CD44^+$ $CD62L^-$ cells were counted by flow cytometry 60 h after stimulation. Representative FACS plot and summary graph are shown. n = 3.

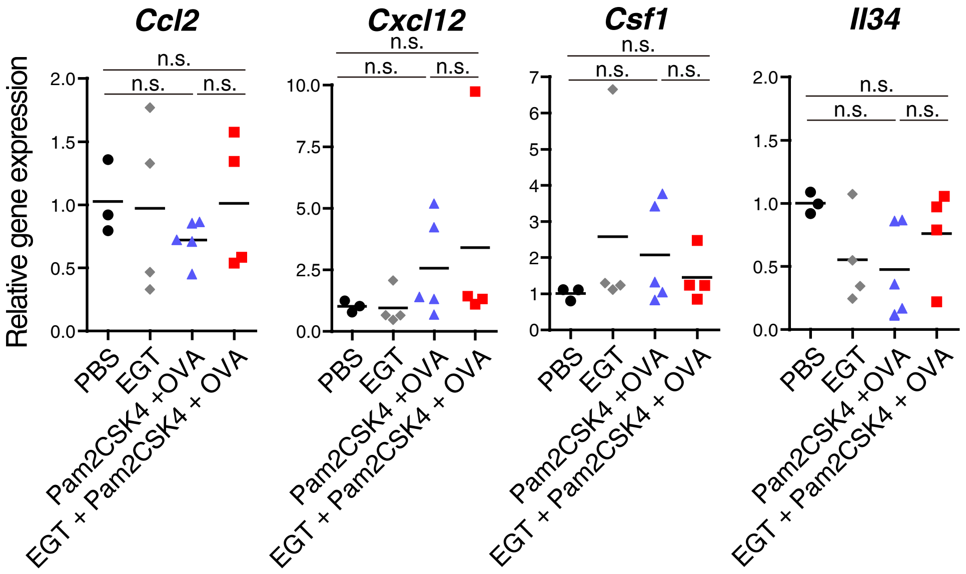

Supplementary Fig. 3. Analysis on RNA expression related to tumor-infiltration or expansion of TAMs. LLC-OVA-implanted WT B6 mice were treated as per Fig. 1A and tumors were harvested at day 18. Then, RNA expression levels of total tumor cells were determined. n = 3-5 mice per group. *P < 0.05. n.s., not significant.

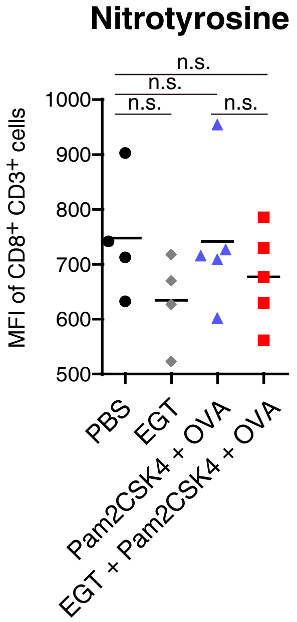

Supplementary Fig. 4. EGT-induced iNOS up-regulation in TAMs is not related to CTL nitraion in tumor. LLC-OVA-implanted WT B6 mice were treated as per Fig. 1A and tumors were harvested at day 18. Total tumor suspension was fix and permeabilized, and then, reacted with anti-nitrotyrosine Ab and PE-conjugated secondary Ab. Mean fluorecent intensity (MFI) of nitrotyrosine was measured by flow cytometry. n = 4-5 mice per group. n.s., not significant.

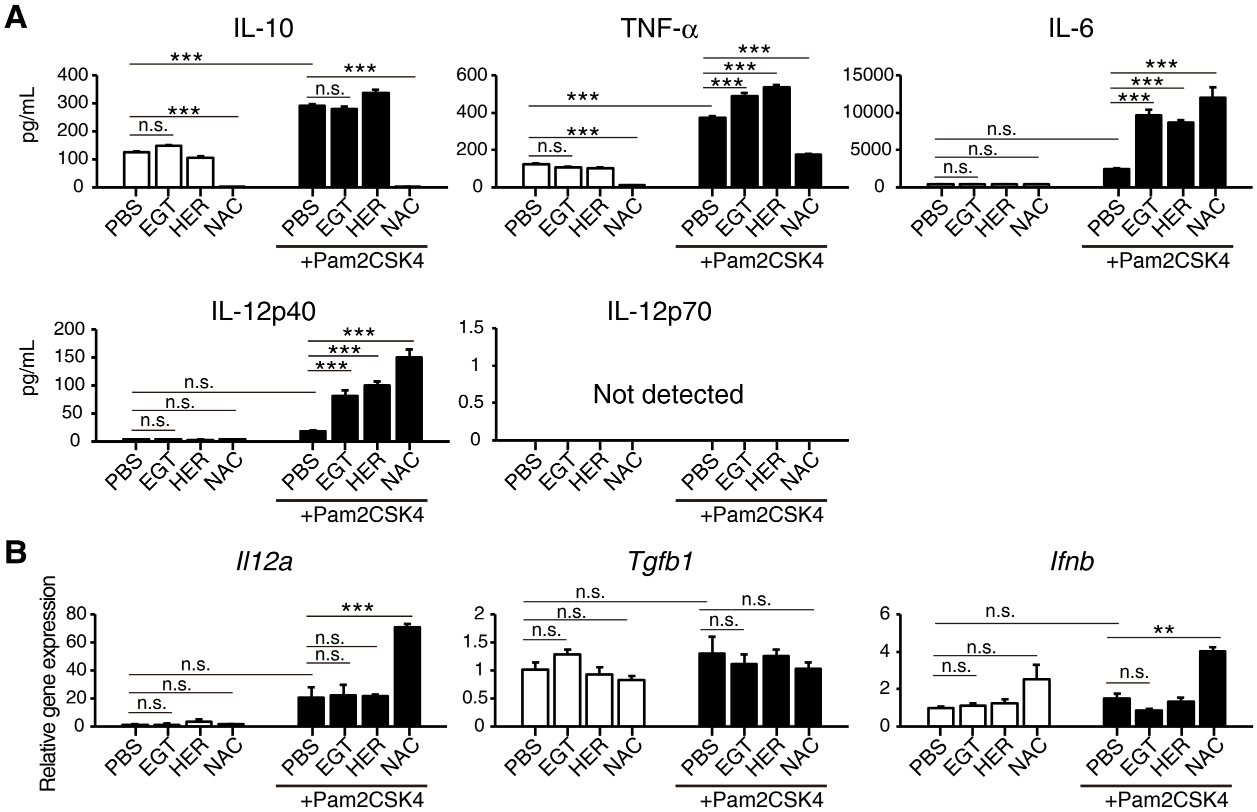

Supplementary Fig. 5. Changes of TAM cytokine production induced by EGT, HER or NAC under TLR2 stimulation. (A,B) Magnetically sorted intratumoral F4/80^{+} cells were treated with or without 10 mM of EGT, HER or NAC for 24h, and then, 50 nM of Pam2CSK4 was added. (A) Culture supernatant was collected 24 h after Pam2CSK4 stimulation. Concentration of cytokines was determined using CBA or enzyme-linked immunosorbent assay (ELISA). ELISA (kits were purchased by BioLegend) was used for quantification of IL-12p40 or Il-12p70, and CBA was conducted to determine other cytokines. n = 3. (B) Total RNA isolated 4 h after Pam2CSK4 stimulation was served to reverse-transcription and qPCR steps. RNA expression normalized to GAPDH was indicated. n = 3. Data are shown as average ± SEM. *P < 0.05, **P < 0.01, *P < 0.001. n.s., not significant.

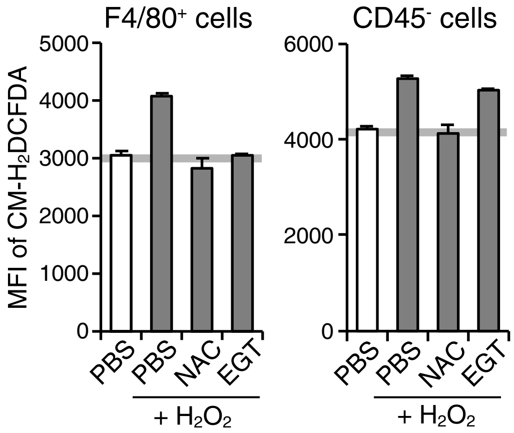

Supplementary Fig. 6. Differential ROS clearance pattern between EGT and NAC in F4/80$^+$ and CD45$^-$ cells. LLC-OVA bearing mice were i.p injected with PBS, EGT (500 μg) or NAC (350 μg) twice with 24 h interval. Tumors were harvested 2 h after last injection. Single cell suspencion was exposed to 1 mM H_2O_2 for 30 min, and then, cells were treated wtih ROS indicator CM-H2DCFDA (Thermo fisher scientific). After 20 min incubation in 37°C and 5% CO2 conditions, cell surface markers were stained by fluorecent Abs and analyzed by flowcytometry. Tumors were pooled from 2 mice per group and data were obtained with duplication (n=2). NAC decreased ROS in both F4/80$^+$ and CD45$^-$ cells. However, EGT cleared ROS in F4/80+ cells but not CD45$^-$ cells.
